# Stressing the limits of capillary blood in anti-doping analysis: perspectives on alkylamine-like stimulants and carbonic anhydrase II inhibitors in result management

**DOI:** 10.3389/fspor.2026.1755735

**Published:** 2026-02-19

**Authors:** Isabelle Karine da Costa Nunes, Mariana Vaz Carneiro, Felipe Alves Gomes de Oliveira, Ana Carolina Dudenhoeffer Carneiro, Carina de Souza Anselmo, Christian Farias Trajano, Monica Costa Padilha, Henrique Marcelo Gualberto Pereira

**Affiliations:** Brazilian Doping Control Laboratory (LBCD), Institute of Chemistry, Federal University of Rio de Janeiro (UFRJ), Rio de Janeiro, RJ, Brazil

**Keywords:** anti-doping, doping control, capillary blood, dried blood spot (DBS), volumetric absorptive microsampling (VAMS), minimum reporting level (MRL), pharmacokinetics, result management

## Abstract

The establishment of an anti-doping rule violation extends beyond the mere detection of a prohibited substance. Current interpretation criteria rely primarily on urinary concentration estimates, which may be misleading due to high interindividual variability or pharmacokinetics characteristics of doping agents. This study investigates the potential of capillary blood collected through volumetric absorptive microsampling (VAMS) as a complementary matrix to urine for supporting results management in doping control. Comparative concentration-time profiling in urine and capillary blood was performed using isometheptene, a stimulant subject to minimum reporting limits (MRLs), and the carbonic anhydrase II inhibitors dorzolamide and brinzolamide as model compounds. For isometheptene, capillary blood detection closely mirrored urinary findings at early time points but exhibited a markedly shorter detection window, supporting improved discrimination between in-competition and out-of-competition administration in cases where urinary concentrations alone may lead to ambiguous interpretation. In contrast, for the long-lasting diuretics brinzolamide and dorzolamide, urinary concentrations showed limited interpretative value, whereas capillary blood analysis provided more consistent detectability, reflecting their strong affinity for red blood cells. However, capillary blood concentrations did not allow unequivocal discrimination between permitted ophthalmic use and prohibited administration routes. Overall, these findings demonstrate that capillary blood analysis can enhance interpretative context of anti-doping result management, particularly for substances with complex pharmacokinetics, and provide a scientific basis for the future establishment of blood-based reporting criteria.

## Introduction

The establishment of an anti-doping rule violation extends beyond the mere detection of a prohibited substance in an athlete's sample (1). In legal and disciplinary proceedings, disputes often focus on the timing, pattern, and circumstances of substance use rather than on analytical detection alone (2, 3). Consequently, anti-doping laboratories are increasingly required to provide interpretative support to Results Management Authorities (RMAs) and Arbitration Courts.

Currently, urine remains the primary biological matrix for doping control analysis. However, for several classes of prohibited substances, urinary concentrations show limited correlation with pharmacological activity at the time of competition, largely due to interindividual variability and substance-specific pharmacokinetic characteristics. These limitations become particularly relevant for substances prohibited in-competition only, where interpretation relies on urinary Minimum Reporting Levels (MRLs) (4–6).

Additional interpretative challenges arise for substances exhibiting pharmacokinetic features such as strong binding to plasma proteins or erythrocytes, reduced renal clearance, or prolonged elimination half-lives. In such cases, urinary concentrations may persist long after pharmacological activity has ceased or, conversely, remain low despite systemic exposure. This limitation is exemplified by the carbonic anhydrase II inhibitors dorzolamide and brinzolamide, which are permitted only via topical ophthalmic administration (4). Their extensive binding to red blood cells results in urinary concentrations poorly correlated with administration timing (7–10).

In this context, capillary blood collected in dried matrix has emerged as a promising complementary sample type for anti-doping analysis and has been discussed and evaluated in different scenarios (6, 11–15). Under the generic term of Dried Blood Spot (DBS), microsampling approaches have been explored for both in-competition and out-of-competition testing scenarios, with particular emphasis on their potential to support result management decisions rather than solely analytical detection.

Volumetric Absorptive Microsampling (VAMS) represents an evolution of the DBS concept, which enable more reliable estimates of doping agent concentration in capillary blood (16–19). Among the commercially available devices, Mitra® and Tasso® systems have been most extensively evaluated in anti-doping research, and comparative assessments of these and other devices are available in the literature (20–22).

From a regulatory perspective, WADA harmonizes analytical requirements and DBS storage procedures through a dedicated technical document (23). The growing interest in DBS had stimulated the development of numerous analytical methods covering various classes of doping agents (24–31). Nevertheless, routine DBS applications in doping control have focused primarily on substances for which detection alone suffices to establish an anti-doping rule violation, such as steroid esters (32–36) and erythropoiesis-stimulating agents (16, 35, 37, 38). In these cases, semi-quantitative or quantitative interpretation is not required. In contrast, the application of VAMS for substances requiring concentration-based interpretation remains underexplored.

This gap highlights a clear opportunity to expand the analytical and interpretative scope of capillary blood testing, particularly for substances with complex pharmacokinetics where urine-based interpretation is limited. However, the diversity of prohibited substances and their distinct pharmacokinetic profiles pose a significant challenge for establishing universal blood-based reporting criteria.

Therefore, this study aims to evaluate the interpretative potential and limitations of capillary blood analysis using VAMS as a complementary matrix to urine in anti-doping result management. Two representative scenarios were investigated: (i) the stimulant isometheptene, subject to urinary MRL criteria and characterized by rapid elimination and high interindividual variability, and (ii) the carbonic anhydrase II inhibitors dorzolamide and brinzolamide, which display prolonged systemic retention and urinary profiles poorly correlated with administration route. By directly comparing urinary and capillary blood concentration-time profiles, this work aims to assess whether capillary blood data can provide additional, scientifically robust support for result interpretation under current anti-doping frameworks.

## Experimental

### Chemicals and reagents

Reference standards were obtained from Cayman Chemical (Ann Arbor, USA), the National Measurement Institute (NMI) (Lindfield, Australia), and Toronto Research Chemicals (North York, Canada). Solvents and reagents, including formic acid, ammonium formate, and methanol were of analytical or HPLC grade (Tedia, Fairfield, USA). Ultrapure water (18 M*Ω*·cm) was produced using a Milli-Q purification system (Millipore, Burlington, MA, USA). Mitra® devices equipped with volumetric absorptive microsampling (VAMS®) tips (30 µL) were purchased from Alllcrom (São Paulo, Brazil). The medications Neosaldina® (300 mg dipyrone, 30 mg isometheptene mucate, and 30 mg anhydrous caffeine), Azopt (brinzolamide 1%), and Cosopt (dorzolamide hydrochloride 2% and timolol maleate 0.5%) were obtained from local retailers.

### Design of administration study

The study protocol was approved by the Ethics Committee of the Federal University of Rio de Janeiro (protocol 40915120.0.0000.5257). Written informed consent was obtained from all participants. Healthy adults of both sexes, aged 20–43 years, were recruited.

Neosaldina® was administered orally to volunteers (*n* = 10) as a single dose of 30 mg isometheptene mucate. For diuretics, Azopt® (brinzolamide) and Cosopt® (dorzolamide) were administered to volunteers as either ocular (*n* = 2 each) one drops in each eye, or two drops orally (*n* = 2 each). Oral administration was conducted using 2 mL of yogurt as a vehicle.

Capillary blood and urine samples were collected at baseline (0 h) and at 2, 5, 7, 24, and 48 h post dose. These time points were selected to capture the absorption phase, peak systemic exposure, and early elimination of the investigated substances. For diuretics, additional late collections were performed at 7 days and 38 days after administration to evaluate long-term urinary detectability, considering their known prolonged systemic retention. Samples from chronic patients undergoing ophthalmic treatment with Azopt® or Cosopt® (one drop twice daily, as prescribed) were collected to provide comparative data representative of repeated therapeutic exposure. Venous blood was drawn 2 h post-dose into K2EDTA-coated tubes (BD Vacutainer®) to determine hematocrit (HCT) and to obtain plasma (for isometheptene) and whole blood (for diuretic analysis), enabling comparison between venous and capillary blood concentrations. Capillary blood samples were collected from the fingertip using Mitra® VAMS devices following the manufacturer's instructions. After samples collection, urine specific gravity was measured and used for concentration adjustment when applicable, in accordance with routine anti-doping practices. Urine samples were storage at −30 °C. Following instructions from the supplier, VAMS were dried for 2 h at room temperature. The dried materials were stored in sealed polyethylene bags containing silica gel at 4 °C until extraction.

### Urine sample preparation

Urine sample volume was fixed at 2.0 mL. Matrix-matched calibration curves were prepared by fortifying blank urine samples from healthy volunteers with isometheptene (2–250 ng/mL) or dorzolamide and brinzolamide (5–200 ng/mL). Deuterated internal standards (IS) (dorzolamide-d5, brinzolamide-d5, and isometheptene-d3) were added to all samples at a final concentration of 50 ng/mL.

For diuretics, liquid–liquid extraction was performed by adding 600 µL of 1 M ammonium formate buffer (pH 4.0) and 1 g NaCl. The pH was adjusted to 4.0 with glacial acetic acid when necessary. For isometheptene, alkaline liquid–liquid extraction was conducted using a Na_2_CO_3_/NaHCO_3_ (1:3) buffer. Subsequently, 5 mL of tert-butyl methyl ether (TBME)/acetate buffer (1:1, v/v) was added, and samples were vortexed and centrifuged (3,000 rpm, 5 min). The organic layer was evaporated under nitrogen at 40 °C and reconstituted in 100 µL of mobile phase mixture (70:30 aqueous: methanolic). UHPLC-MS/MS analyzed urine extracts.

### VAMS sample preparation

For calibration and quality control, venous whole blood collected in K_2_EDTA tubes was fortified with isometheptene (5–50 ng/mL) or with dorzolamide/brinzolamide (5–200 ng/mL). Blank and fortified blood samples were applied to Mitra® VAMS tips and processed as described below.

For extraction, VAMS tip were detached and transferred to microtubes containing 5 μL of IS working solution contained either isometheptene-d3 or brinzolamide-d5/dorzolamide-d5 (50 ng/mL each) and 400 μL of extraction solvent (methanol/acetonitrile/2% aqueous acetic acid, 1:1:1, v/v/v). Samples were sonicated for 30 min at 30 °C, centrifuged (17,000 g, 5 min). Supernatants were evaporated under nitrogen at 40 °C, and residues were reconstituted in 100 µL of mobile phase (80:20, aqueous:methanolic) prior to UHPLC–MS/MS analysis.

### UHPLC–MS/MS analysis

Analyses were performed using a Dionex UHPLC system coupled to a TSQ Quantiva triple-quadrupole mass spectrometer (Thermo Fisher Scientific, Bremen, Germany) equipped with electrospray ionization source operating in positive ion mode. Chromatographic separation was achieved on a Syncronis C8 column (1.7 µm, 50 mm × 2.1 mm, Thermo Fisher Scientific) maintained at 40 °C. The mobile phases consisted of (A) water with 5 mM ammonium formate, (B) methanol, and (C) acetonitrile, all of which contained 0.1% formic acid. Injection volume was 10 µL.

For urine samples containing brinzolamide and isometheptene, the gradient program for mobile phase B was as follows: 15% (0 min) → 80% (17.5 min), held for 0.5 min, returned to 15% (18.1 min), and equilibrated until 20 min, with a flow rate of 0.3 mL/min. For dorzolamide urine samples, phase B started at 1%, increased to 5% (1.5 min), then to 90% (7 min), and was held for 2 min. It was then returned to 1% (9.1 min) and equilibrated for 15 min at a flow rate of 0.4 mL/min.

For VAMS samples, the gradient for phase C began at 10%, increased to 35% (25 min), then to 80% (30 min), and was held at 100% (32 min). It then returned to 10% (32.1 min) and equilibrated for 35 min at 0.3 mL/min.

The ion transfer tube and vaporizer were set to 350 °C and 400 °C, respectively. Spray voltage was static; sheath, auxiliary, and sweep gas flows were 40, 10, and 2 (arbitrary units). Selected reaction monitoring (SRM) transitions, collision energies, and precursor/product ions as follows: Isometheptene [M + H]^+^ *m/z* 142 → *m/z* 69 (16); *m/z* 111 (10); *m/z* 58 (13). Dorzolamide [M + H]^+^ *m/z* 325 → *m/z* 135 (30), *m/z* 151 (30), *m/z* 219 (30). Brinzolamide [M + H]^+^ *m/z* 384 → *m/z* 217 (30), *m/z* 163 (30), *m/z* 195 (30). For quantification purposes, the ISTD-normalized peak obtained from the extracted ion chromatograms of the first precursor/product ion pairs above mentioned in each analyte was adopted. Brinzolamide-d5 was monitored in urine using the mass transition [M + H]^+^ *m/z* 389 → *m/z* 136 (43). To improve specificity, the product ion was changed to *m/z* 281 due to an endogenous interference. Dorzolamide-d5 was monitored using transition [M + H]^+^ *m/z* 330 → *m/z* 135 (30), and isometheptene-d3 were monitored using the transition [M + H]^+^ *m/z* 145 → *m/z* 69 (15) independently of the matrix.

Peak width was set to 20 s, cycle time to 2 s, and Q1/Q3 resolution to 0.7 FWHM. Data acquisition and processing were performed using TraceFinder 4.2 software (Thermo Fisher Scientific).

### Method validation

Blank samples from different volunteers were used to build the set of replicates during the validation. The procedures used for semi-quantitative determination of the analytes in urine and capillary blood were validated following the analytical features:

- Selectivity: Evaluated by analyzing drug-free urine and capillary whole-blood (VAMS) samples (*n* = 10 for each matrix) to verify the absence of endogenous or matrix-related interfering signals.
- Limit of Quantification (LOQ): Each analyte were spiked in the analytical matrix from 1 to 4 ng/mL. Defined as the lowest concentration fulfilling WADA identification criteria (39) and a signal-to-noise ratio > 10.
- Intra-day precision: Estimated based on analyses performed within the same analytical day and determined at three concentration levels (5, 20, and 200 ng/mL) using spiked urine and capillary blood samples. It was expressed as relative standard deviation (RSD, %).
- Inter-day precision: Estimated based on analyses performed on different analytical days by different analysts, using the same three concentration levels (5, 20, and 200 ng/mL). It was expressed as RSD (%).
- Linearity: Assessed using matrix-matched calibration curves prepared in the matrix over the concentration range of 5–200 ng/mL. Calibration curves were constructed by regression analysis of ISTD-normalized peak area ratios vs. concentration.
- Carryover: Evaluated by injecting blank samples after fortified ones at 400 ng/mL (urine) and 200 ng/mL (VAMS). The absence of detectable analyte signals in the blank samples indicated that no carryover occurred under the applied analytical conditions.
- Matrix effect: Evaluated by comparing analyte signal areas obtained from post-extraction spiked samples with those from the respective mobile phase in equivalent concentrations.

For calibration, quality control, and intraday/interday precision experiments, venous whole blood collected in K_2_EDTA tubes was fortified at defined concentration levels and subsequently applied onto Mitra® VAMS devices to generate dried blood samples (23).

## Results and discussion

### Methods validation

The methods for semi-quantitative analysis were validated and the results were tabulated in Supplementary Material 1.

The absence of interfering signals confirmed the specificity of the methods, and LOQs were 2–3 ng/mL for isometheptene, 4 ng/mL for dorzolamide, and 2–4 ng/mL for brinzolamide, depending on the matrix (urine or VAMS). Those values were established considering a double criterion: i) signal-to-noise ratio of 10 and ii) meeting identification criteria (39). Since matrix from different volunteer were adopted, the same values were considered the methods Limit of Identification (LOI). Linearity were acceptable having as criteria *R*^2^ ≥ 0.99. Ion suppression or enhancement was observed (from 60% to 108%) depending on the analyte and matrix. The results were accepted considering the use of deuterated IS for each analyte. Intra-day and inter-day did not exceed 20% in the low concentration evaluated (5 ng/mL). For both parameters, imprecision was lower than 10% at 200 ng/mL. These results were considered acceptable considering no-threshold substances. The absence of detectable analyte signals in the blank samples after programed injection indicate absence of carry-over.

### Concentration estimates

To ensure reliable concentration estimates, VAMS devices and isotope-labelled internal standards were employed were included to minimize potential matrix effects arising from interindividual variability in blood and urine composition. For urine, concentration values were adjusted to the specific gravity. The hematocrit level could potentially impact in the diffusion properties of the capillary blood in the card/paper sampling devices. Theoretically, the adoption of Mitra®/VAMS eliminates this impact (26, 40). Capillary and venous blood concentrations obtained at the first sampling time point were comparable, and no systematic bias attributable to hematocrit values (37.4–47.0%) was observed.

### Alkylamines—stimulant sub-class

The stimulant class comprises hundreds of substances with wide structural and pharmacokinetic diversity (41). The subclass of alkylamines has drawn attention due to the high incidence of dietary supplement contamination. In Brazil, isometheptene is of special concern given its widespread availability in over-the-counter headache medications and the prevalent culture of self-medication (42). There are no reports in the literature on the evaluation of alkylamines by capillary blood. Furthermore, the class of stimulants is not included in the Testing Menu defined by dedicated DBS Technical (23). These factors motivated the selection of isometheptene as a model compound in an exploratory investigation.

### Isometheptene—urinary vs. VAMS profiling comparison

**Figure 1:**
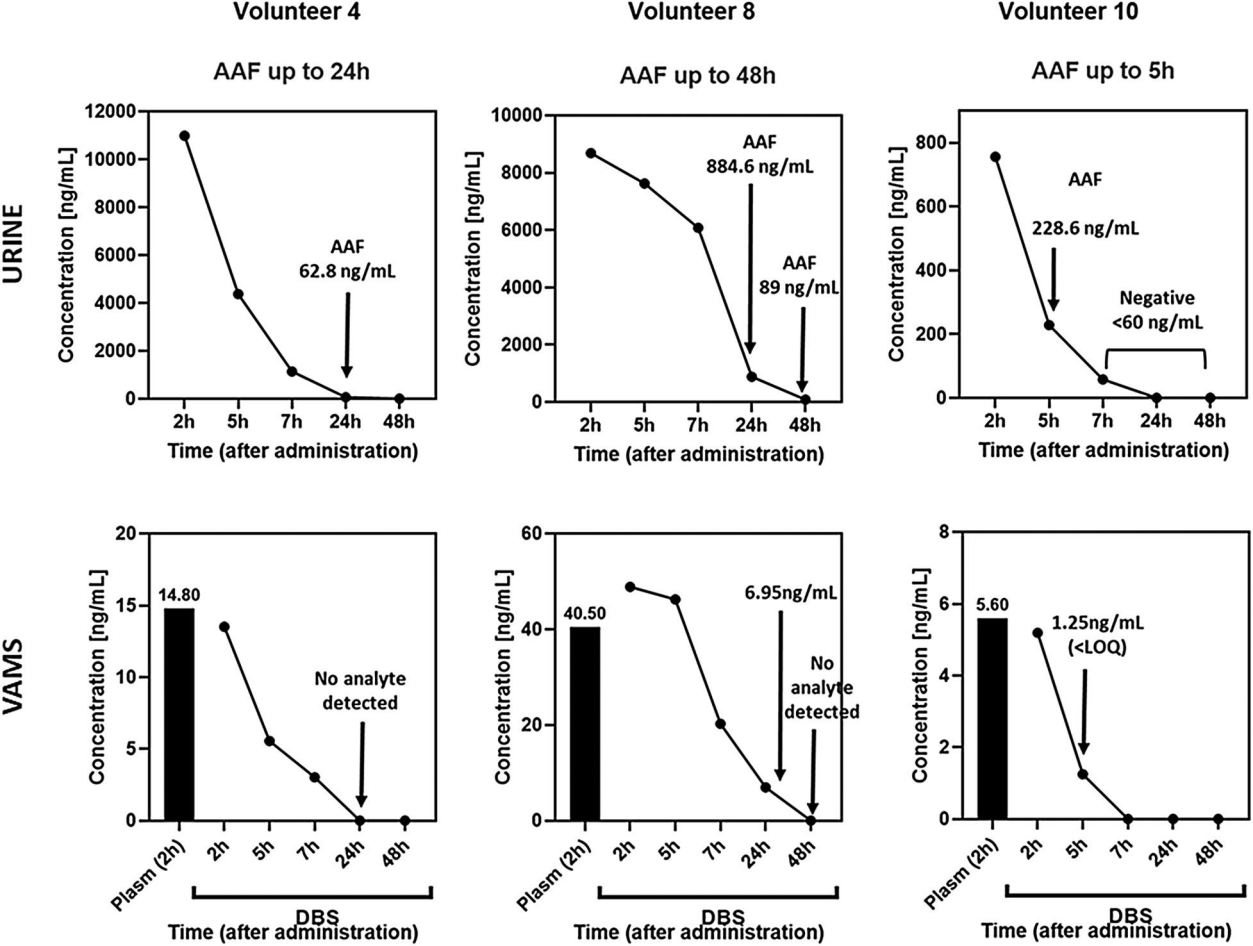
Isometheptene urinary and capillary blood concentrations profiles. The analyte was not detected in the blood of volunteers 4 and 8 at 24 h and 48 h, respectively. However, in urine samples, analyte concentrations above the MRL were observed at the same point after administration. For Volunteer 10, the graph indicates a urinary concentration of 228.6 ng/mL at the 5 h time point, while in capillary blood, the analyte was below the LOQ.

Following a single oral dose of isometheptene, all volunteers presented detectable urinary concentrations at 2 h post-administration, corresponding to individual Cmax values and confirming rapid absorption. Marked interindividual variability was observed, with urinary Cmax values ranging from approximately 12,000 ng/mL to less than 800 ng/mL (Figure 1). In all volunteers, the concentrations in this collection point exceeded the MRL (50 ng/mL) by at least two orders of magnitude, clearly indicating an Adverse Analytical Finding (AAF), even though some values fell outside the validated linear range.

The urinary excretion profile demonstrated a rapid decline in isometheptene concentrations. In 70% of volunteers, levels fell below the MRL within 7 h of administration, indicating that therapeutic in-competition use could theoretically result in a negative finding. Volunteer 04 presented a urinary isometheptene concentration of 68 ng/mL at 24 h, and Volunteer 08 exhibited 89 ng/mL at 48 h post-dose, both cases corresponding to potential AAFs (Figure 1).

Although no MRL is currently defined for blood/capillary blood samples under WADA regulations, the comparison of matrices provides valuable insight. Isometheptene was detectable in capillary blood samples from all volunteers (2 h post-administration), mirroring the urinary data. As in urine, Cmax in capillary blood exhibited high interindividual variability, likely reflecting pharmacokinetic differences that are typically unavailable in anti-doping contexts. Therefore, only estimated concentrations were considered, in accordance with the routine protocol.

Isometheptene was undetectable in capillary blood samples collected 7 h' post-dose in all volunteers who also tested negative for isometheptene in urine at the same time window. In four cases, blood concentrations were detectable, but below the LOQ. Notably, 37% of urine samples that would produce an AAF had corresponding capillary blood concentrations below the LOQ.

Volunteers 04 and 08, whose urinary concentrations would trigger AAFs in a hypothetic out of competition period administration, illustrate this dissociation. For Volunteer 04, isometheptene was undetectable in capillary blood 24 h post-dose, with levels already below 5 ng/mL at 7 h. As expected, volunteer 08 showed a longer detectability of isometheptene in capillary blood. However, concentrations were approximately 7 ng/mL at 24 h and undetectable at 48 h after administration. These values are substantially lower than hypothetical DBS cut-offs proposed for other stimulants as cocaine (20 ng/mL), benzolecgonina (75 ng/mL) and amphetamine (20 ng/mL) in the literature (6), suggesting the suitability of these value ranges also for isometheptene.

The pronounced interindividual variability highlights the challenge faced by RMAs if only urinary MRLs were used as an interpretation tool and underscores the potential risk for athletes using isometheptene-containing medications. Similar concerns have been reported for other stimulants, including amphetamine, methylphenidate, and modafinil, which are also listed as doping agents (43). Based on these findings, a conservative washout period should be strongly recommended following administration.

The occurrence of AAFs based on urinary concentrations in the absence of detectable levels in capillary blood raises questions about the assertiveness of the MRL values established for urine. If the stimulant is not detected in blood, it likely indicates that the compound is either no longer pharmacologically active or has been rapidly eliminated. Consequently, urinary concentration alone may not accurately reflect the physiological impact of the substance at the time of sample collection, critical point considering in competition controls. These findings reinforce that urine-based MRLs have inherent limitations, and incorporating capillary blood through VAMS analysis could provide a more accurate basis for RMA (43).

Indeed, the shorter detection window observed in capillary blood samples suggests improved discrimination between in-competition and out-of-competition administration and may strengthen result adjudication in cases involving alleged inadvertent exposure, such as supplement contamination (11, 43, 44).

Considering the interindividual variability observed in urinary excretion, establishing a universal MRL for an entire stimulant class is particularly remains challenging, as similarly observed for glucocorticosteroids class (45). The detection or non-detection of a doping agent in capillary blood may provide complementary evidence for RMAs in doping cases involving substances prohibited in-competition only.

Finally, the structural and physicochemical diversity within the stimulant class underscores the importance of additional comparative studies. To our knowledge, this is the first investigation focused on the alkylamine subclass, providing new insights into its pharmacokinetic behavior and implications for anti-doping result interpretation.

#### Inhibitors of carbonic anhydrase II as a doping agent

Dorzolamide ranks among the Top 3 diuretics reported in anti-doping controls, accounting for approximately 14% of all diuretic findings, while brinzolamide appears within the Top 5 (9%) according to the most recent WADA statistics (46). Although ophthalmic administrations are permitted and may be declared on doping control forms, no analytical criterion currently exists to determine the route of administration (47). Consequently, any detected urinary concentration constitutes an AAF, leaving route assessment to RMAs. While non-ophthalmic pharmaceutical preparations are uncommon, oral administration remains feasible.

The issue is further complicated by the fact that up to 80% of a topically administered ocular dose may reach systemic circulation via the nasopharyngeal route (48), leading to systemic carbonic anhydrase inhibition. Although animal studies have suggested the potential use of metabolic patterns to distinguish oral from ophthalmic administration (47), no validated criterion exists in humans, and the impact of administration route on urinary excretion remains uncertain (49). Previous reports have described wide ranges of urinary concentrations following chronic administration, limiting the interpretative value of urine alone for results management (49–51). These limitations motivated the investigation of capillary blood as a complementary matrix.

#### Dorzolamide/brinzolamide—urinary profiling

**Figure 2:**
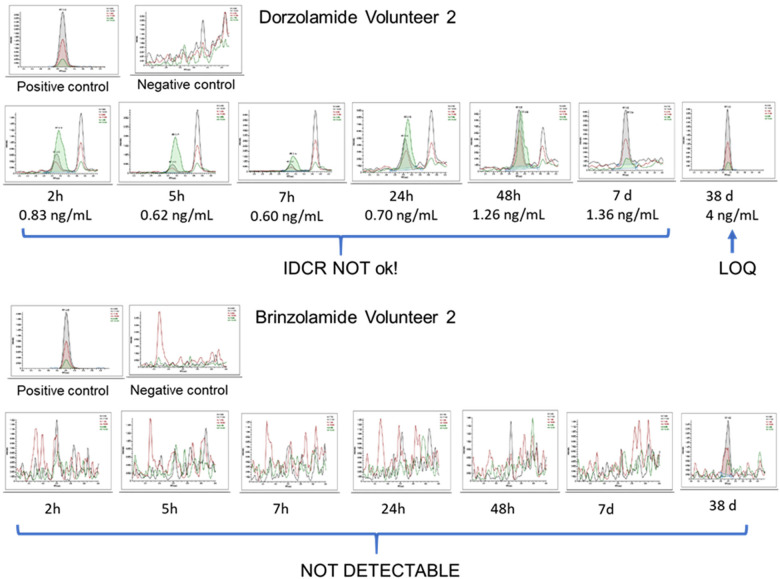
Representative chromatograms of the concentration assessment in urine after the oral administration of dorzolamide (upside) show the presence of peaks at the analyte's retention time across different collection times. However, the identification criteria were only met in the late collected sample evaluated (38 days). Brinzolamide (downside): Not detected, even in the late collection.

Urine samples from treated volunteers showed very low dorzolamide concentrations up to 7 days following both oral (prohibited) and ophthalmic (permitted) administration. Both routes produced similar urinary detectability and concentration patterns, precluding route discrimination when targeting the parent compound alone. This is consistent with topical administration resulting in systemic absorption, as mentioned above. A representative urinary profile following oral administration is shown in Figure 2. The late urine collection performed approximately five weeks (38 days) after the administration resulted in an estimated dorzolamide concentration close to the LOQ (4 ng/mL). This late detectability aligns with pharmacokinetic data describing a high affinity for CA II and extensive binding to red blood cells (10) as well as with empirical observations from sample routine samples analysis (7). Although detection of dorzolamide in early urine samples allows monitoring of the parent compound in competition in cases of hypothetical oral administration, the controlled administration study confirms that urinary results alone do not allow differentiation between oral and ophthalmic routes. Proposals for alternative approaches that allow for such a diagnosis are welcome (47).

For brinzolamide, urinary profiling posed an even greater challenge. Despite similar pharmacokinetics and an elimination half-life of approximately 111 days (Azopt—NDA 20-816/S-009), the parent compound was not detected within the first 48 h of collection, or even in the sample collected 7 days after administration (Figure 2), severely limiting any criterion based solely on urinary parent compound concentration.

Based on these excretion profiles, additional anti-doping samples reported as AAF by the Brazilian Anti-Doping Laboratory (LBCD) between 2020 and 2023 were evaluated. During this period, 19 AAFs involving dorzolamide or brinzolamide were reported, of which 84% originated from out-of-competition testing, suggesting repeated rather than single-dose exposure. Dorzolamide accounted for 83% of the cases.

**Figure 3:**
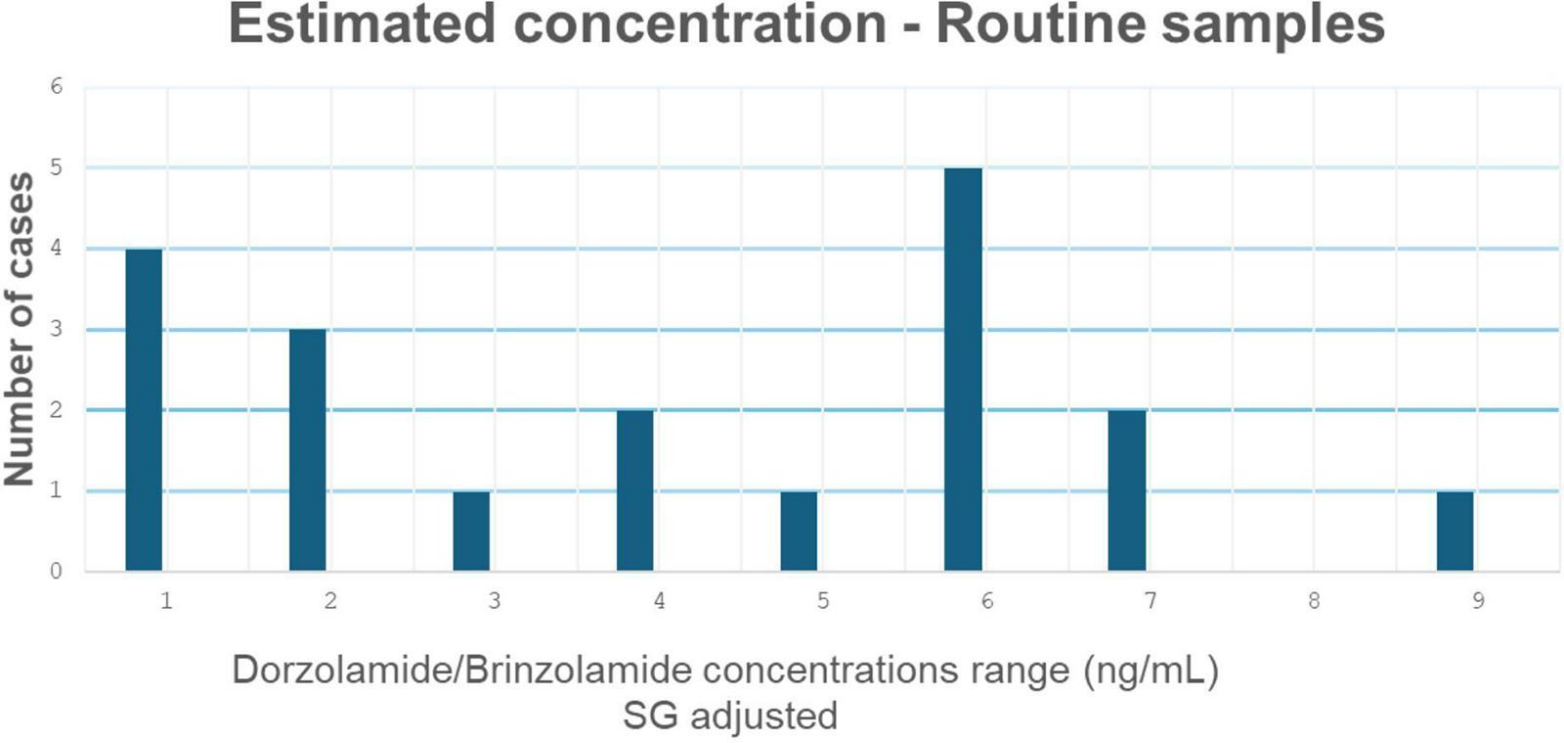
Distributions of estimated concentrations of dorzolamide/brinzolamide in samples reported as adverse analytical findings—urine matrix. Concentration adjusted by specific gravity. Dorzolamide and brinzolamide are, here, treated as a single population.

Specific gravity–adjusted urinary concentrations ranged from 2 to 435 ng/mL (Figure 3). Only one case presented concentrations near the LOQ, potentially reflecting discontinued use with residual trace levels, due to prolonged elimination, or very recent administration, consistent with the controlled study results.

Urinary concentrations measured in chronic ophthalmic users ranged from 19 ng/mL to 932 ng/mL. overlapping with values observed in athlete samples. Although the number of chronic users evaluated was limited (*n* = 2), this overlap supports the hypothesis that most routine AAFs are associated with continuous administration rather than incidental exposure.

Notably, 63% of the AAFs involved mixed martial arts (MMA) athletes, a discipline organized by weight categories (52, 53). Whether this reflects legitimate ophthalmologic treatment or intentional use for potential diuretic effects cannot be determined analytically so far. Nevertheless, this association should be considered by Results Management Authorities.

#### Inhibitors of carbonic anhydrase II—urinary vs. capillary blood profiling comparison

**Figure 4:**
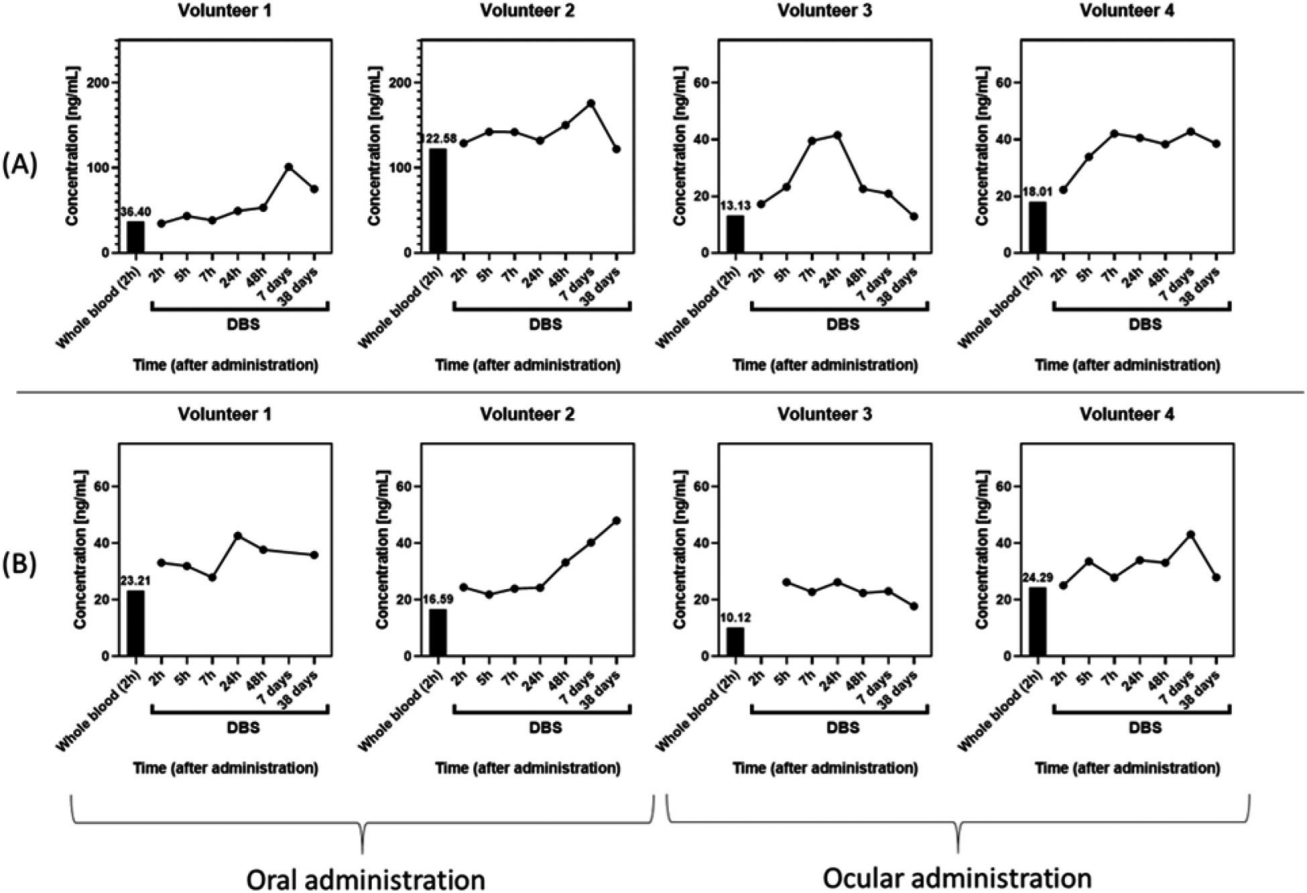
Whole blood (black bars) and VAMS concentrations profiles after diuretics administration. **(A)** Dorzolamide. **(B)** Brinzolamide.

In contrast to urine, capillary blood analysis yielded markedly different results for dorzolamide and brinzolamide, consistent with their strong affinity for red blood cells. Following dorzolamide administration in four volunteers (two oral and two ophthalmic), capillary blood concentrations were observed after oral dosing, suggesting increased systemic exposure. Conversely, brinzolamide exhibited less interindividual variability across administration routes (Figure 4B).

Although, further studies are required for definitive conclusions, these results indicate that occasional or short-term use at therapeutic doses may not be detectable in urine, emphasizing the potential of capillary blood to provide complementary information on systemic exposure for doping control, particularly for substances with these pharmacokinetic characteristics.

Accordingly, for CA II inhibitors listed by WADA, capillary blood may enhance interpretative accuracy in anti-doping testing. In the current regulation, brinzolamide and dorzolamide should not be included as part of the analytes in the DBS Multi Class Menu, according to TD2026DBS (23). Nevertheless, the findings presented here should be interpreted as exploratory and hypothesis-generating, contributing to the scientific basis for future evaluations of capillary blood applicability in result management.

Despite improved detectability, capillary blood results alone, targeting the parent compounds, do not allow unequivocal discrimination between permitted ophthalmic use and deliberate misuse and should therefore be regarded as complementary rather than definitive evidence for RMAs.

The high incidence of dorzolamide/brinzolamide findings in a specific sport modality also warrants further investigation. From a practical perspective, considering their limited diuretic efficacy (if demonstrated) and predominant ophthalmologic use, the continued inclusion of these substances in the WADA Prohibited List should be critically re-evaluated.

## Data Availability

The original contributions presented in the study are included in the article/Supplementary Material, further inquiries can be directed to the corresponding author/s.
