# Supplementary material for: Stressing the limits of capillary blood in anti-doping analysis: perspectives on alkylamine-like stimulants and carbonic anhydrase II inhibitors in result management
Source: Front Sports Act Living. 2026 Feb 19;8:1755735. doi: 10.3389/fspor.2026.1755735 (PMC12961188; doi:10.3389/fspor.2026.1755735)
Supplement: Supplementary file 1 [file Datasheet1.pdf]

Material Supplementary 1 – Validation results. Urine and capillary blood (CB) measurements.

	Isometheptene		Dorzolamide		Brinzolamide		Details
	Urine	Capillary Blood	Urine	Capillary Blood	Urine	Capillary Blood	
Selectivity	√	√	√	√	√	√	-
LOQ (ng/mL)*	2	3	4	4	2	4	S/N >10 / IDCR OK
Intra-day precision	5.8%<br>4.5%<br>5.9%	6.6%<br>5.5%<br>6.9%	15.5%<br>3.9%<br>7.4%	11.6%<br>11.57%<br>6.5%	7.4%<br>5.5%<br>3.7%	10.1%<br>13.6%<br>6.2%	at 5 ng/mL<br>at 20 ng/mL<br>at 200 ng/mL
Inter-day precision	10.6%<br>6.2%<br>8.1%	8.6%<br>5.8%<br>8.1%	13.0%<br>6.5%<br>9.5%	15.5%<br>11.7%<br>8.8%	17.6%<br>4.8%<br>4.2%	15.6%<br>13.7%<br>6.7%	at 5 ng/mL<br>at 20 ng/mL<br>at 200 ng/mL
Linearity (day 1)<br>(day2)<br>(day 3)	0.9940<br>0.9960<br>0.9989	0.9987<br>0.9917<br>0.9778	0.9998<br>0.9999<br>0.9995	0.9978<br>0.9952<br>0.9966	0.9987<br>0.9995<br>0.9994	0.9981<br>0.9966<br>0.9937	From 5 ng/mL to 200 ng/mL
Carry over	0.0%	0.0%	0.0%	0.0%	0.0%	0.0%	Urine – 400 ng/mL<br>CB – 200 ng/mL
Matrix effect	60%	109%	102%	108%	80%	101%	n = 3

√ = 100% of the blank samples analyzed resulted in a Negative Finding

*Estimated as the lowest concentration detectable with a signal-to-noise ratio > 10 and IDCR criteria fulfilled.
